# Preclinical study of pachyman inducing ferroptosis against ovarian cancer: Biological targets and underlying mechanisms

**DOI:** 10.1002/fsn3.3534

**Published:** 2023-06-29

**Authors:** Bihui Li, Yanyan Deng, Xuqiang Lin, Xiaowei Wan, Jiaqi Liu

**Affiliations:** ^1^ Department of Oncology The Second Affiliated Hospital of Guilin Medical University Guilin China; ^2^ Department of Gynecology, Guigang City People's Hospital The Eighth Affiliated Hospital of Guangxi Medical University Guigang China; ^3^ Department of Pathology, Guigang City People's Hospital The Eighth Affiliated Hospital of Guangxi Medical University Guigang China; ^4^ Key Laboratory of Environmental Pollution and Integrative Omics, Guilin Medical University Education Department of Guangxi Zhuang Autonomous Region Guilin China

**Keywords:** bioinformatics findings, ferroptosis, malignant features, ovarian cancer, Pachyman

## Abstract

Ferroptosis has gained extreme purpose in targeting cancer treatment. *Poria cocos Wolf*, a traditional Chinese herb, has potential anticancer properties, but the action and mechanism against ovarian cancer remain undetailed. Pachyman (*Poria cocos* polysaccharides) refers to the pharmacologically bioactive ingredients rich in *Poria cocos*. This study aimed to identify the potent actions and the network mechanisms of pachyman against ovarian cancer through preclinical analysis. Online‐accessible database or platform was employed to predict candidate genes and core targets associated with ferroptosis in pachyman against ovarian cancer. Enrichment analyses were used to characterize the functional action and signaling mechanism in pachyman to treat ovarian cancer. Molecular docking imitation was conducted for verification of core target proteins. Network analysis uncovered that there were 30 mutual and 13 core genes targeting ferroptosis in pachyman and/against ovarian cancer, and additional enrichment analysis characterized that these core genes may act synergistically through multiple biological processes and molecular pathways associated with ferroptosis, including anti‐inflammatory action, immunoregulation, and microenvironment modulation. The strongest affinities in core target proteins between pachyman and sarcoma (SRC), signal transducer, and activator of transcription 3 (STAT3) were further validated using molecular docking method. In conclusion, pachyman may induce antiovarian cancer potentials via regulating ferroptosis‐associated biological functions and pharmacological mechanisms based on current bioinformatics findings. We reason that pachyman, the beneficial nutraceuticals, may be used clinically for future application in ovarian cancer treatment.

## Graphical abstract

Our bioinformatics findings reveal the biological actions, key genes, and signaling pathways targeting ferroptosis in Pachyman treating ovarian cancer potentially. This study will better contribute to complete understanding of Pachyman‐mediated network mechanisms in treating ovarian cancer. Notably, identified interactions of drug and core gene may lay a theoretical foundation for the research and development of ovarian cancer drugs targeting ferroptosis.

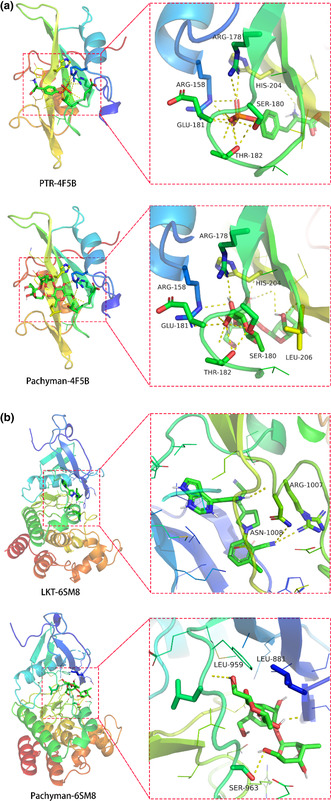

## 1 INTRODUCTION

Ovarian cancer is a malignant, invasive, and metastatic disease that characterizes unmanaged morbidity and increased death rate globally (Stewart et al., 2019). It is epidemiologically reported that the prevalence and fatality of ovarian cancer is mounting recently in Asia (Shanmughapriya et al., 2013), leading to elevated economic burden in developing countries. The pathogenesis of ovarian cancer is complicated. The underlying relevancy is ferroptosis, a unique cell death that can control cell proliferation in human cancers (Lei et al., 2022). Increasing reports display the potency via inducing ferroptosis for cancer treatment, especially to suppress invasive neoplasm that occurs in drug resistance to chemotherapy (Liang et al., 2019). Some approved medicines, such as sorafenib and sulfasalazine, can trigger ferroptosis and inactivate neoplastic growth. Additionally, ferroptosis‐based dysfunctions may generate immune inflammation suppression in neoplastic microenvironment, eventually promoting tumor growth (Chen et al., 2021) or inducing drug tolerance (Friedmann Angeli et al., 2019). Therefore, further exploration of substitute cancer treatment targeting ferroptosis is urgently needed in clinical practice. There is still no curative treatment for ovarian cancer, and existing therapy against ovarian cancer is effective insufficiently before potent invasion (Eisenhauer, 2017). In addition, long‐period chemotherapy for ovarian cancer may result in adverse effects, including nausea, vomiting, alopecia, diarrhea, constipation, and neuropathy (Frey et al., 2017). Traditional Chinese medicine, such as natural ingredients from medicinal plants, has interestingly paid close attention to therapeutic potentials for human disorders (Xiang et al., 2019). It is a preclinical description that soy isoflavones may exert benefits in treating sleep deprivation‐induced cognitive deficits through reducing oxidative impairment and inhibiting neuroinflammation (Lu et al., 2022). Plant cannabis oil may improve reserpine‐caused fibromyalgia in vivo and serve as a potential alternative (Raposo et al., 2022). Some extracts from *Meripilus giganteus* exhibit to play antiproliferative actions against leukemic cells in vitro (Lenzi et al., 2018). Cruciferous vegetables are rich in bioactive cancer‐protective compounds. Increasing evidences have suggested that daily intake of sulfurous vegetables can contribute to reduction of tumorigenesis, including colorectal cancer (Mitra et al., 2022). Natural flavonoids may elicit double actions for the drug combination in enhancing chemotherapy efficacy and reducing side effects for chemotherapy against colorectal cancer (Fernández et al., 2021). Diallyl disulfide is a main organosulfur ingredient, a naturally occurring bioactive compound in garlic. It is found experimentally that DADS exerts anticancer potential against many human tumors, including ovarian cancer (Mitra et al., 2022). *Poria cocos* has been applied historically in folk treatment against ailments in Asia over 2000 years (Ríos, 2011). *Poria cocos* extracts, including polysaccharides, triterpenoids, and sterols, may be preclinically found with effective immunomodulation, antitumor, and anti‐inflammation activities (Li, Ma, & Zhang, 2019). Pachyman, also termed as *Poria cocos* polysaccharides, refers to a cluster of functional nutraceuticals, characterized by anticancer signatures, such as hepatic carcinoma (Qin et al., 2021). In addition, carboxymethylated pachyman is reported with antiovarian cancer action targeting ferroptosis and the underlying mechanism is achieved through inhibition of nuclear factor erythroid 2‐related factor 2 (Nrf2)/heme oxygenase‐1 (HO‐1) signaling pathway (Jing et al., 2022). Recent evidences have indicated that gut microbiome and the metabolites may serve as cancer promotor or inhibitor (Ağagündüz et al., 2023). Some natural polysaccharides are found with potential anticancer benefits through regulating gut microbiota and immunological function (Guo et al., 2021). Other findings show that carboxymethyl pachyman may relieve intestinal mucositis via modulation of intestinal microflora in tumor‐bearing mice (Wang et al., 2018). Moreover, polysaccharides can be considered as the promising natural ingredients with high safety and nontoxic side effect, including pachyman (Zhang et al., 2023). However, further study of pachyman in antiovarian cancer action and mechanism associated with ferroptosis remain unreported currently. The network‐characterizing approach, such as network pharmacology, can be an advantageous strategy to uncover pharmacological features, including drug repurposing, medicine‐target prediction, and novel bioactive ingredient discovery (Yang et al., 2022). *In silico* identification of the bioactive compound−target protein binding affinity by using molecular docking combination is an emerging methodology for revealing drug−target interactions (DTIs) (Ferreira et al., 2015). Thus, this study tended to ascertain the antiovarian cancer characteristics of pachyman targeting ferroptosis via identifying optimal genes and enriched core genes. And then, the core DTIs in pachyman against ovarian cancer targeting ferroptosis were subsequently determined with molecular docking analysis for characterizing core proteins. Our present study will contribute to a better understanding of the antiovarian cancer targets and mechanisms associated with ferroptosis in pachyman (the natural nutraceuticals) before future clinical application.

## 2 METHODS

### 2.1 Collecting and selecting target genes

To begin with, Swiss TargetPrediction (http://www.swisstargetprediction.ch/), PharmMapper (http://www.lilab‐ecust.cn/pharmmapper/), and SuperPred (https://prediction.charite.de/) databases were used for collecting pachyman‐linked target genes, respectively. Secondly, FerrDb (http://www.zhounan.org/ferrdb) and GeneCards (http://www.genecards.org) databases were applied for ferroptosis‐associated target genes. Thirdly, GeneCards and Online Mendelian Inheritance in Man (OMIM, http://omim.org) were employed to select ovarian cancer‐related target genes. All data were identified on October 2022 after removing duplication. And then, the data were used for additional Venn‐map analysis to determine the correlative target genes in pachyman against ovarian cancer targeting ferroptosis.

### 2.2 Producing DTIs and acquiring core target genes

DTI‐related network of correlative target genes was constructed by using Cytoscape software (version 3.8.2) (https://cytoscape.org/) and the data was processed from String database (https://string‐db.org/). The confidence score cutoff was set at 0.7. The core target genes were determined through the standard parameters, in which the upper limit of the filtering range was the maximum degree in parametrical data, and the lower limit was the median degree of freedom, as in our previous report (Liu, Meng, et al., 2022).

### 2.3 Gene ontology (GO) and Kyoto encyclopedia of genes and genome (KEGG) enrichment annotation analysis

ClusterProfiler and GOplot in R package and cluster profile were used to build and enrich GO and KEGG pathway annotations after target genes were retrieved. Among these tests, the *p*‐value cutoff and *q*‐value cutoff were set at .05, accordingly. Top 20 annotations from GO and KEGG enrichment analysis in pachyman against ovarian cancer targeting ferroptosis were visualized using R package in the form of bubble and circle diagrams, respectively. The more procedures were detailed as previously described (Chen et al., 2017).

### 2.4 Molecular docking pattern

Molecular docking imitation was used to implement via the Autodock Vina program (https://vina.scripps.edu/) for identifying the core proteins in pachyman against ovarian cancer. The chemical structure of pachyman was downloaded from the PubChem database (https://pubchem.ncbi.nlm.nih.gov/). Other three‐dimensional structures of SRC and STAT3 core proteins were obtained from Protein Data Bank (PDB; https://www.rcsb.org/) with PDB‐ID: 4F5B and PDB‐ID: 6SM8. The Autodock Tool was applied to prepare the structures before ligand docking. Molecular docking data were determined by referencing root‐mean‐square deviation (RMSD). Other details in screening were shown in previous report (Liu, Guo, et al., 2022).

## 3 RESULTS

### 3.1 Target genes in pachyman, ovarian cancer, and ferroptosis

**FIGURE 1:**
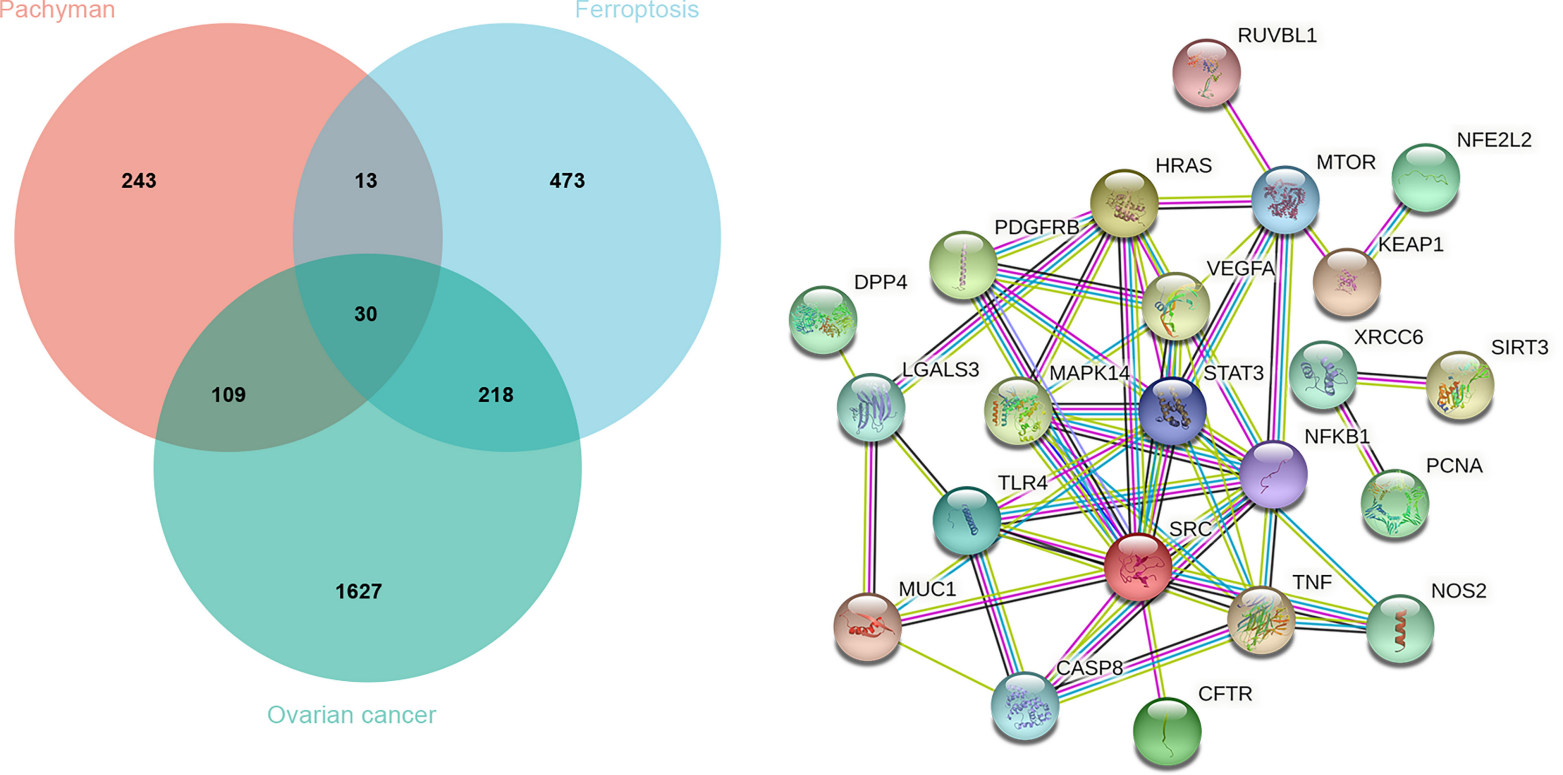
Venn diagram analysis exhibited the potential target genes in pachyman, ferroptosis, and ovarian cancer, respectively. Additional 30 interaction target genes among pachyman, ferroptosis, and ovarian cancer were identified and further constructed in a connecting network.

Aided by open‐source database analysis, we identified all 395 pachyman‐linked target genes, 1984 ovarian cancer‐related target genes, and 734 ferroptosis‐associated target genes. As shown in Venn diagram and drug−target interactions (Figure 1), candidate genes and shared genes were ascertained in the intersections among pachyman, ovarian cancer, and ferroptosis. In addition, additional 30 intersection genes identified from common genes were displayed in networks.

### 3.2 Core target genes associated with ferroptosis

**FIGURE 2:**
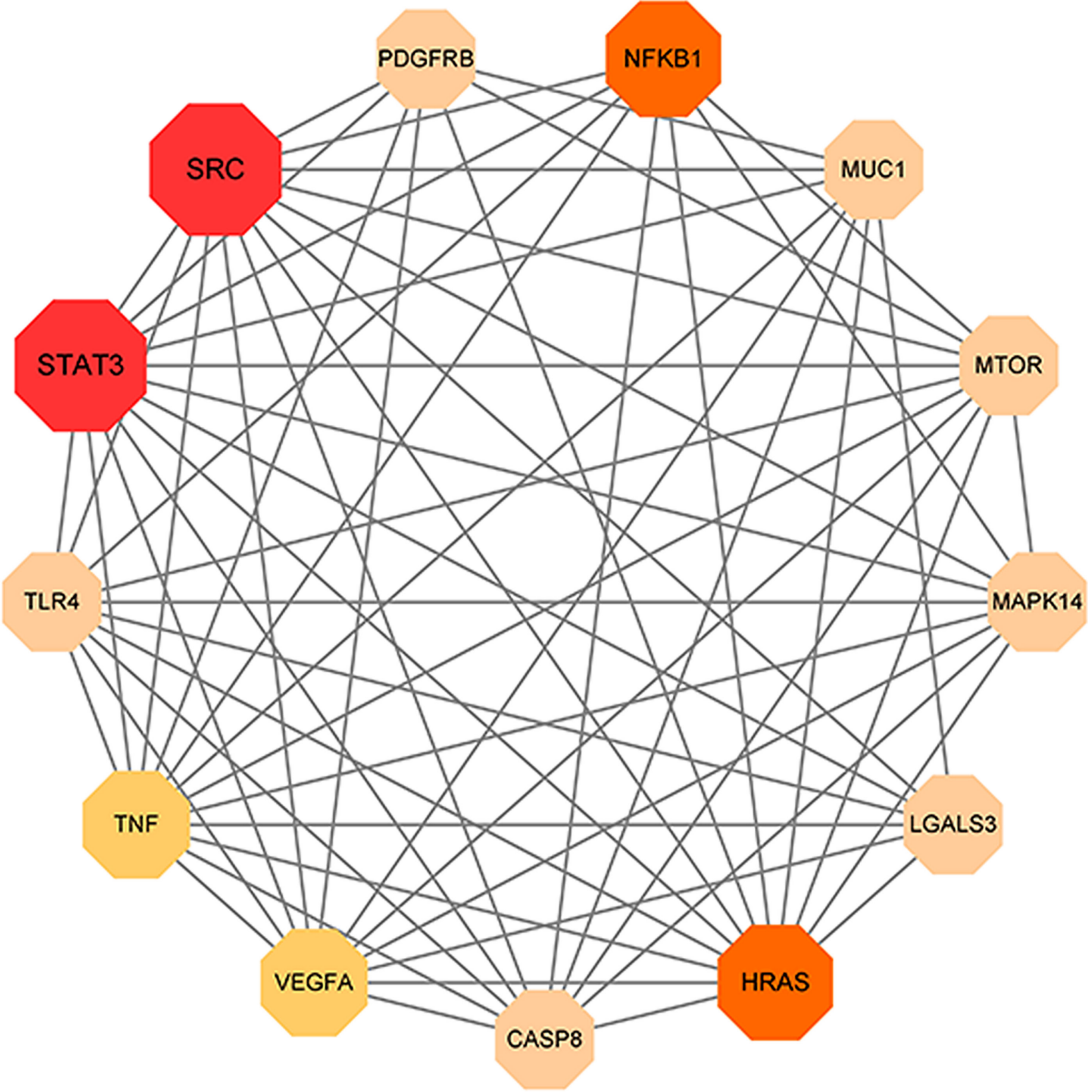
These intersection targets from preliminary bioinformatics analysis were redetermined for identification of core target genes in pachyman against ovarian cancer associated with ferroptosis, as highlighted in a connecting network.

Topological parameter analysis was used to identify core target genes related to ferroptosis in pachyman in the treatment of ovarian cancer. The median degree of freedom in screening of core target genes was set as 4, and the maximum degree of freedom was set as 11. As a result, the range of core gene screening was set as 4−11. Ultimately, all 13 core target genes associated with ferroptosis in pachyman against ovarian cancer were identified, namely, SRC, STAT3, and kappa light polypeptide gene enhancer in B‐cells 1 (NFKB1), Harvey rat sarcoma viral oncogene homolog (HRAS), tumor necrosis factor (TNF), vascular endothelial growth factor A (VEGFA), toll‐like receptor 4 (TLR4), mitogen‐activated protein kinase 14 (MAPK14), rapamycin (MTOR), caspase‐8 (CASP8), Mucin 1 (MUC1), galectin 3 (LGALS3), and platelet‐derived growth factor receptor beta (PDGFRB) (Figure 2).

### 3.3 Enrichment annotation results

**FIGURE 3:**
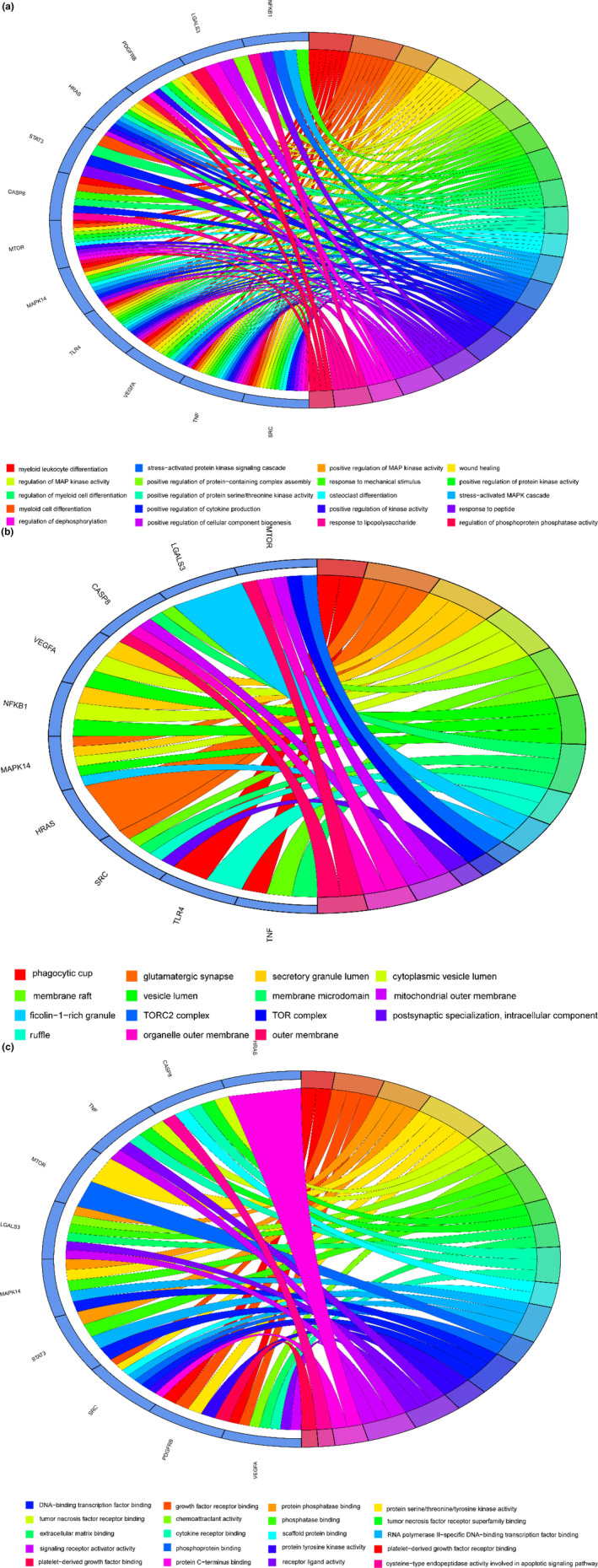
For further annotation analysis of these core target genes, GO enrichment findings indicated that the potent pharmacological actions in pachyman treating ovarian cancer associated with ferroptosis, as detailed in top biological processes (a), top cellular components (b), top molecular functions (c).

**FIGURE 4:**
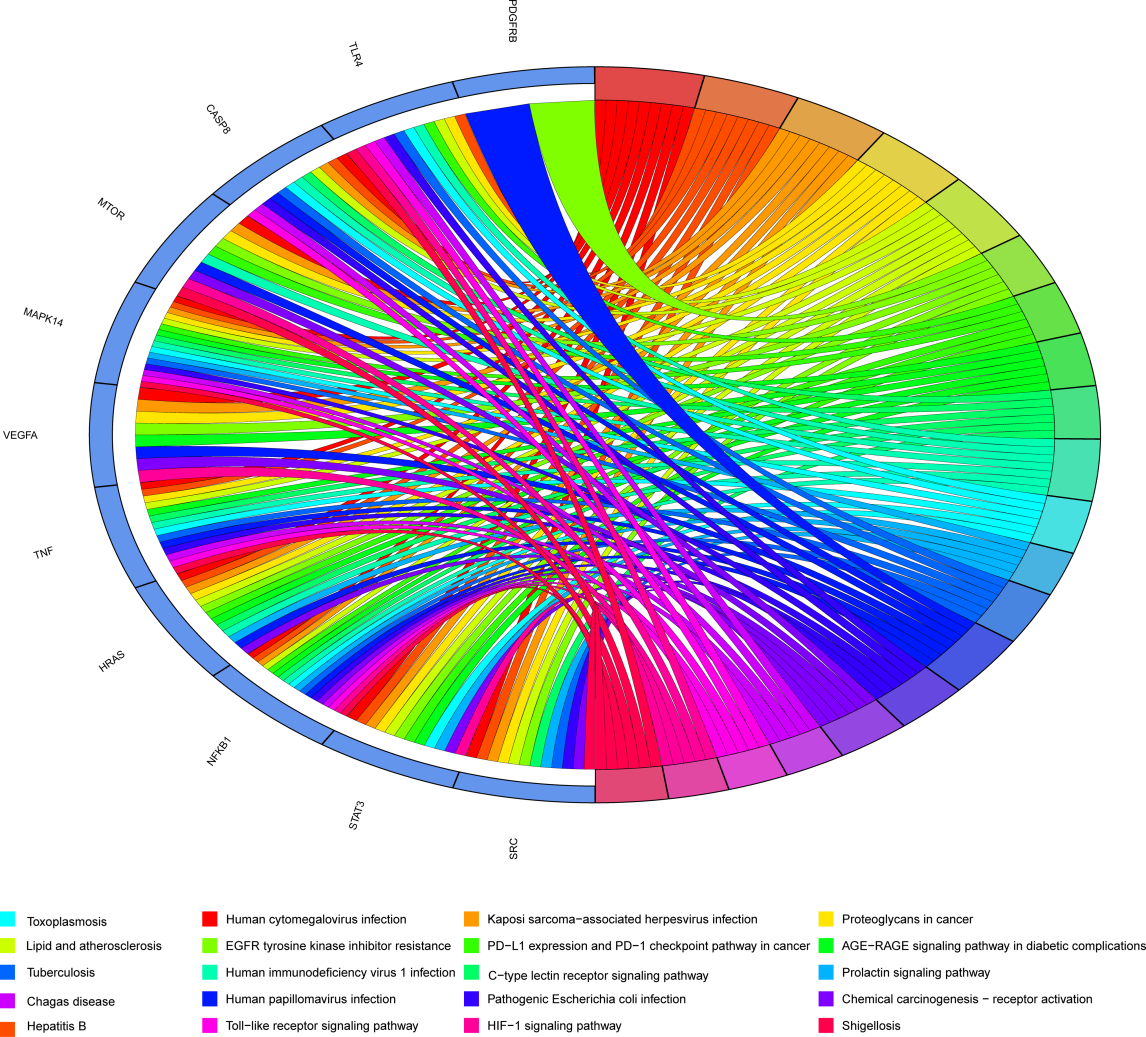
KEGG enrichment findings of core target genes for actions in top signaling pathways aimed to reveal the antiovarian cancer mechanisms associated with ferroptosis exerted by pachyman. *p* < .05 in all reported pathways.

**FIGURE 5:**
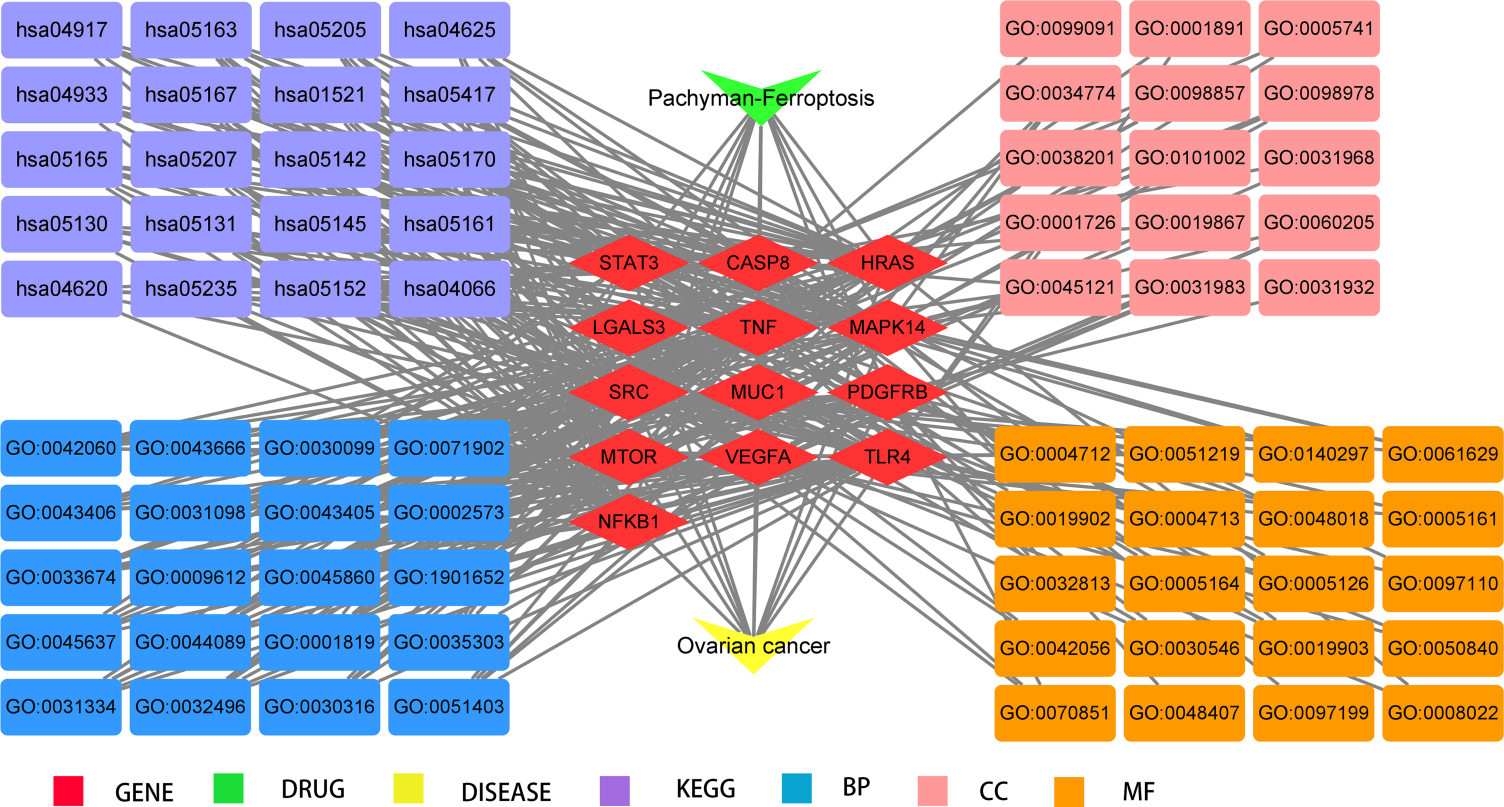
Together with current network pharmacology data, we concluded that all bioinformatics findings were holistic integrated in the network chart associated with ferroptosis, as characterized in pachyman−target−pathway−ovarian cancer network.

The GO‐enriched categories from core target genes distinguished biological processes (BPs), cellular components (CCs), and molecular functions (MFs). The top 20 BPs of core target genes are displayed in Figure 3a. Enrichment results indicated that pachyman possibly contributed to antiovarian cancer actions associated with BPs targeting ferroptosis were mainly involved in myeloid leukocyte differentiation, myeloid cell differentiation, positive regulation of MAP kinase activity, wound healing, regulation of MAP kinase activity, positive regulation of protein‐containing complex assembly, response to mechanical stimulus, positive regulation of protein kinase activity, regulation of myeloid cell differentiation, positive regulation of protein serine/threonine kinase activity. Antiovarian cancer actions of pachyman associated with CCs were largely involved in phagocytic cup, glutamatergic synapse, secretory granule lumen, cytoplasmic vesicle lumen, membrane raft, vesicle lumen, membrane microdomain, ruffle, ficolin‐1‐rich granule, and rapamycin complex‐2 (TORC2) (Figure 3b). Antiovarian cancer actions of pachyman associated with MFs were primarily involved in platelet‐derived growth factor receptor binding, growth factor receptor binding, protein phosphatase binding, protein serine/threonine/tyrosine kinase activity, tumor necrosis factor receptor binding, chemoattractant activity, phosphatase binding, tumor necrosis factor receptor superfamily binding, extracellular matrix binding, and cytokine receptor binding (Figure 3c). In addition, KEGG categories were enriched with false discovery rate (FDR) <0.05 in core target genes, resulting in 116 pathways. Top KEGG‐enriched pathways targeting ferroptosis were chiefly involved in human cytomegalovirus infection, hepatitis B, Kaposi sarcoma‐associated herpesvirus infection, proteoglycans in cancer, lipid and atherosclerosis, epidermal growth factor receptor (EGFR) tyrosine kinase inhibitor resistance, programmed death ligand 1 (PD‐L1) expression and PD‐1 checkpoint pathway in cancer, advanced glycation end product (AGE)‐receptor for AGE (RAGE) signaling pathway in diabetic complications, C‐type lectin receptor signaling pathway, human immunodeficiency virus 1 infection, toxoplasmosis, prolactin signaling pathway, tuberculosis, human papillomavirus infection, pathogenic *Escherichia coli* infection, chemical carcinogenesis‐receptor activation, Chagas disease, toll‐like receptor signaling pathway, hypoxia‐inducible factor‐1 (HIF‐1) signaling pathway, and shigellosis (Figure 4). Briefly, a holistic network diagram of drug−component−target−pathway interactions in pachyman against ovarian cancer is highlighted in Figure 5.

### 3.4 Molecular docking data

**FIGURE 6:**
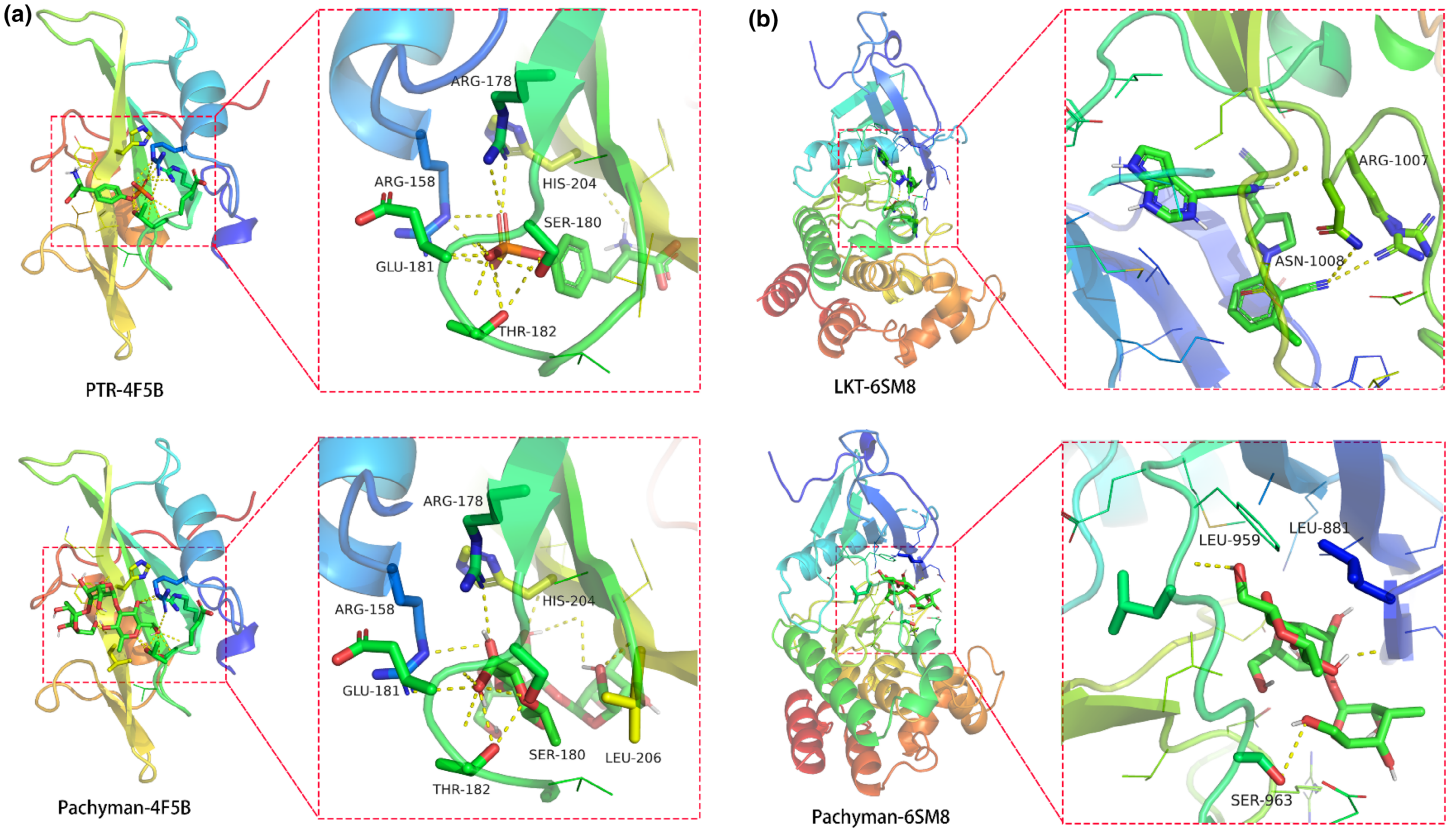
*In silico* analysis showed three‐dimensional crystal structures of SRC, STAT3 being docked by pachyman. (a) Biologically docking of pachyman to SRC (PDB ID: 4F5B). (b) Biologically docking of pachyman to STAT3 (PDB ID: 6SM8). Current data *in silico* indicated that these core proteins might be potential drug targets in pachyman against ovarian cancer.

As revealed in the data of three‐dimensional crystals, pachyman might effectively dock with SRC (4F5B) and STAT3 (6SM8), and the free docking energy and RMSD calculated for each binding model were −5.5 kJ/mol (RMSD: 1.767 Å) and −8.1 kJ/mol (RMSD: 2.181 Å), respectively. Pachyman binding amino acid residues (4F5B) via forming hydrogen bonds detailed in LEU‐206 (3.1 Å), HIS‐204 (2.9 Å), ARG‐178 (3.3 Å), ARG‐158 (3.2 Å), SER‐180 (3.2 Å), GLU‐181 (3.1 Å), and THR‐182 (2.8 Å) (Figure 6a). In addition, pachyman binding amino acid residues (4F5B) via forming hydrogen bonds detailed in LEU‐959 (3.3 Å), SER‐963 (3.4 Å), and LEU‐881 (2.0 Å) (Figure 6b). These molecular docking findings indicated strong interactions between pachyman with SRC and STAT3 and the potential pharmacological targets against ovarian cancer.

## 4 DISCUSSION

Ovarian cancer refers to the malignant tumor growing on the ovary, in which more than 75% of affected cases are primary ovarian cancer and the other parts are metastasized tumors (Doubeni et al., 2016). Current clinical application of maintenance of chemotherapy for treating ovarian cancer is prescribed with poly (adenosine diphosphate‐ribose) polymerase (PARP) inhibitors. However, these medicines result in a potential difference in therapeutic outcome based on breast cancer susceptibility gene (BRCA) mutations and unselected patterns (Armstrong, 2018). Exploring substitute therapeutics to suppression of the malignant development of ovarian cancer remains a priority for all oncologists. Nowadays, there are no modern drugs that have been proven with substantial evidence for disease‐curing effectiveness in malignant ovarian cancer. Accumulating evidences suggest that ferroptosis plays potential physiological roles in tumor suppression and immunity, oxido‐reduction homeostasis, and cell‐to‐cell interactions, as well as oncogenic and tumor suppressor signaling (Jiang et al., 2021). Thus, ferroptosis is of large potential in modulating proliferation, metastasis, and tumor stem cell resistance (Valashedi et al., 2022). Therefore, targeting ferroptosis for managing cell death may be a potentially promising strategy in malignancy treatment, including ovarian cancer. *Poria cocos Wolf* has been found with potential efficacy for adjuvant therapy in chronic disorders, such as cancers (Nie et al., 2020). Existing studies support that pachyman, the plant polysaccharides rich in *Poria cocos*, exerts beneficial immunoregulation (Chu et al., 2012) and potent anticancer benefits (Li, He, et al., 2019). For an in‐depth revealment of antiovarian cancer network mechanisms, further investigation aims to detail the pharmacological effects of bioactive pachyman components. In our present study, *in silico* bioinformatics analysis was applied to unlock the existent limitations of beneficial valuation of traditional ingredients. Validated by the natural components of pachyman as well as diversified pharmacological targets against cancers, experimental assessment is impracticable relatively to reveal comprehensive molecular mechanisms behind therapeutic effectiveness of these natural polysaccharides. Herein, based on network pharmacology combined with molecular docking analyses may contribute to further detail by identifying pharmacological targets and mechanisms in bioactive pachyman in the treatment of ovarian cancer. Network pharmacology report exhibited that a total of 30 candidate target genes among pachyman−ovarian cancer−ferroptosis were obtained after determination by database tools. Among these targets, part of these genes has been referenced for pathophysiology functions in ovarian cancer, such as VEGFA (Jang et al., 2017). After accomplishing consequent analysis, all core target genes were completely identified, including SRC, STAT3, NFKB1, HRAS, TNF, VEGFA, TLR4, MAPK14, MTOR, CASP8, MUC1, LGALS3, and PDGFRB. Furthermore, GO enrichment data from these core genes suggested that pachyman might play antiproliferative effects against ovarian cancer through modulating cell differentiation, kinase activity, receptor binding capabilities, and chemoattractant activity. It is reported recently that kinase inhibitors can be used to clinically treat ovarian cancer to suppress cell proliferation (Katopodis et al., 2019). Increasing evidences show the pathological relevance between receptor expression and ovarian cancer's oncogenesis, and some hormone receptors are considered to be associated with cell proliferation, invasion, and stemness (Chung et al., 2021). Ferroptosis‐initiating therapy may exhibit promising clinical potential for suppressing the development of cell proliferation or differentiation in ovarian cancer (Yung et al., 2022). KEGG enrichment findings based on current core genes indicated universally shared signaling pathways across EGFR tyrosine kinase inhibitor resistance, PD‐L1 expression and PD‐1 checkpoint pathway in cancer, C‐type lectin receptor signaling pathway, prolactin signaling pathway, chemical carcinogenesis‐receptor activation, toll‐like receptor signaling pathway, HIF‐1 signaling pathway. Targeted therapy for ovarian cancer can choose inhibition of misadjusted angiogenesis and uncontrolled DNA repair for better management of cell cycle, and signaling pathways in tumors (Grunewald & Ledermann, 2017). Dysfunction of DNA repair can cause tumor genomic instability and tumor formation. And PD‐1/PD‐L1 molecular pathway can induce tumor immune escape, and this signaling may be a potential pharmacological target for immunotherapy against ovarian cancer (Zhang et al., 2021). Studies have reported that many conventional molecular pathways and functional processes are found with involvement in ferroptosis, in which ferroptosis can exert the key actions in the genesis, development and metastasis of ovarian cancer (Zhao et al., 2022). Notably, pachyman‐medicated antioxidative and anti‐inflammatory properties have been crucial to attention in the previous report (Zhao et al., 2020). Together, pachyman may play an overall effect concerning different biological functions and multiple molecular pathways that may function via targeting ferroptosis to treat ovarian cancer potentially. The component−target−pathway networks revealed that regulator mechanisms were positive interactions between multiple targets and signaling pathways in pachyman treating ovarian cancer through relating to ferroptosis. In addition, most of the abovementioned targets were determined again for *in silico* validation. As a result, determined docking scores among SRC, STAT3, and pachyman were the relatively highest affinities among all tested core proteins. Despite interactions between pachyman and SRC, STAT3 is still unreported directly in preclinical experiments. Both SRC and STAT3 are regarded as potential targets in ovarian cancer treatment (Giordano et al., 2021; Liang et al., 2020). These biological effects of STAT3 may be related to targeting ferroptosis manner (Qiang et al., 2020). Based on current bioinformatics data, we might extrapolate that SRC and STAT3 were core pharmacological targets in pachyman treating ovarian cancer through regulating ferroptosis. And preclinical antiovarian cancer mechanisms exerted by pachyman are revealed accordingly. However, owing to the absence of validation by essential data, visible experiment is still needed to conduct for proving these bioinformatics findings in the present study.

## 5 CONCLUSION

Taken together, our bioinformatics findings reveal the biological actions, key genes, and signaling pathways targeting ferroptosis in pachyman treating ovarian cancer potentially. This study will better contribute to promote an understanding of pachyman‐mediated pharmacological mechanisms in treating ovarian cancer. Notably, identified interactions of pachyman and core gene may lay a theoretical foundation for the research and development of ovarian cancer drugs targeting ferroptosis.

## AUTHOR CONTRIBUTIONS

**Bihui Li:** Data curation (equal); formal analysis (equal); investigation (equal); methodology (equal); validation (equal). **Yanyan Deng:** Data curation (equal); formal analysis (equal); methodology (equal); resources (equal); software (equal). **Xuqiang Lin:** Data curation (equal); formal analysis (equal); resources (equal); software (equal). **Xiaowei Wan:** Methodology (equal); resources (equal); software (equal). **Jiaqi Liu:** Investigation (equal); project administration (equal); resources (equal); supervision (equal); visualization (equal); writing – original draft (equal); writing – review and editing (equal).

## FUNDING INFORMATION

This research is partially supported by the second affiliated hospital of Guilin Medical University and health key discipline construction project.

## CONFLICT OF INTEREST STATEMENT

The authors confirm that there are no known conflicts.

## Data Availability

Research data are not shared.
